# Clonal Dissemination of Clinical Carbapenem-Resistant *Klebsiella pneumoniae* Isolates Carrying *fosA3* and *bla*_*KPC*–2_ Coharboring Plasmids in Shandong, China

**DOI:** 10.3389/fmicb.2021.771170

**Published:** 2021-12-17

**Authors:** Yingying Hao, Xuguang Zhao, Cui Zhang, Yuanyuan Bai, Zhen Song, Xinglun Lu, Ran Chen, Yaoyao Zhu, Yueling Wang

**Affiliations:** ^1^Department of Clinical Laboratory, Shandong Provincial Hospital, Cheeloo College of Medicine, Shandong University, Jinan, China; ^2^Department of Clinical Laboratory, Shandong Provincial Hospital Affiliated to Shandong First Medical University, Jinan, China; ^3^Department of Clinical Laboratory, The People’s Hospital of Shouguang City, Shouguang, China; ^4^Department of Clinical Laboratory, Feicheng Hospital of Shandong Guoxin Yiyang Group, Feicheng, China

**Keywords:** *Klebsiella pneumoniae*, fosfomycin, carbapenem resistant, *fosA3*, *bla*_*KPC*–2_

## Abstract

Treatment strategies of infection by carbapenem-resistant *Klebsiella pneumoniae* (CRKP) are limited. Fosfomycin, a broad-spectrum antibiotic, has attracted renewed interest in combination therapy to fight *K. pneumoniae* infections. However, reports on fosfomycin-resistant *K. pneumoniae* are increasing. Among the 57 CRKP strains, 40 (70.2%) were resistant to fosfomycin. Thus, whole-genome sequencing and bioinformatics analysis were conducted to reveal molecular characteristics of fosfomycin-resistant *K. pneumoniae*. Twenty-three isolates coharbored *fosA^kp^* and *fosA3*, with *K. pneumoniae* carbapenemase (KPC)-producing ST11-KL64-wzi64-O2 (*n* = 13) and ST11-KL47-wzi209-OL101 (*n* = 8), the predominating clonal groups, while *fosA3* was not detected in isolates carrying class B carbapenemase genes. Twenty-two (out of 26) ST11-KL64 strains were positive for *rmpA2*, of which 12 carried *fosA3*. Four of the 23 *fosA3*-positive isolates could successfully transfer their fosfomycin-resistant determinants to *Escherichia coli* J53Azi*^R^*. All four strains belonged to ST11-KL47 with the same pulsed-field gel electrophoresis profile, and their transconjugants acquired fosfomycin, carbapenem, and aminoglycoside resistance. A 127-kb conjugative pCT-KPC-like hybrid plasmid (pJNKPN52_KPC_fosA) coharboring *fosA3*, *bla*_*KPC*–2_, *bla*_*CTX–M*–65_, *bla*_*SHV*–12_, *rmtB*, and *bla*_*TEM*–1_ was identified. ST11-KL64 and ST11-KL47 *K. pneumoniae*, with higher resistance and virulence, should be critically monitored to prevent the future dissemination of resistance.

## Introduction

Carbapenem-resistant *Klebsiella pneumoniae* (CRKP) poses a serious challenge in clinical medicine (Zhou et al., 2020). CRKP can cause bloodstream infections that are difficult to treat, with dramatically high hospital mortality rates of 55.8% (Xiao et al., 2020). In 2017, the WHO published a list of critical priority pathogens, where CRKP was placed in the Priority 1 group (Ekwanzala et al., 2019). Drug resistance is spreading rapidly across microbial species; however, new antibiotics are not discovered as frequently. Therefore, reassessing the utility of and the mechanism of drug resistance among microbes against long-established antibiotics is necessary (Vardakas et al., 2016).

Fosfomycin, discovered in 1969, is a broad-spectrum antimicrobial agent targeting peptidoglycan synthesis (Liu et al., 2020). It is effective against carbapenem-resistant Enterobacterales (CRE) and extended-spectrum β-lactamase-producing (ESBL) Enterobacterales and CRE, *in vitro*, and is approved for the management of systemic infections in Spain, Germany, and France (Mączyńska et al., 2021). In China, intravenous fosfomycin has been employed in the treatment of systemic infectious diseases since the 1990s. However, resistance has gradually increased over the past decade; in China, 80% of *K. pneumoniae* carbapenemase-producing *K. pneumoniae* (KPC-KP) strains are resistant to fosfomycin, which is much higher than in other countries (Huang et al., 2021).

Routine susceptibility test for fosfomycin in non-*Escherichia coli* Enterobacterales is not feasible (Elliott et al., 2019; Mączyńska et al., 2021). Clinical and Laboratory Standards Institute (CLSI) recommends glucose-6-phosphate (G6P)-supplemented broth microdilution and agar dilution for accurate susceptibility testing. Even tests like Kirby–Bauer Disk Diffusion (DD) and *E*-tests cannot yield reliable results and screen the fosfomycin resistance phenotype. Moreover, the epidemiology of fosfomycin resistance in clinical CRKP isolates from Shandong, China, is unclear.

Three mechanisms of fosfomycin resistance have been reported (Huang et al., 2021). Mutations in *glpT* and *uhpT* genes affect L-a-glycerophosphate and hexose-6-phosphate uptake, respectively. Mutations in MurA binding site, most notably Asp369Asn and Leu370lle, can also confer fosfomycin resistance. However, the most potent mechanism of resistance is drug hydrolysis by various chromosomal or plasmid-borne fosfomycin hydrolases, including FosA (*fosA2*, *fosA3*, *fosA4*, *foskp96*, *fosA^kp^*, and *fosA7*), *fosB*, *fosC*, and *fosX* (Liu et al., 2020).

Although the high fosfomycin resistance rate among KPC-KP was considered predominantly caused by clonal dissemination, horizontal transfer of *fosA3*-encoding plasmids among KPC-KP was also documented; in particular, the emergence of conjugative plasmids carrying a combination of the *fosA3* and *bla*_*KPC*–2_ genes could accelerate the spread of antibiotic resistance (Jiang et al., 2015; Chen et al., 2019; Zhang et al., 2019). However, little is known regarding the prevalence of plasmid-mediated *fosA3* and *bla*_*KPC*–2_ co-dissemination among KPC-KP.

In this study, we aimed to elucidate the molecular epidemiology of fosfomycin resistance among clinical CRKP isolates in China and to determine their genetic lineages. One of the self-transmissible plasmid pJNKPN52_KPC_fosA harboring *fosA3*, *bla*_*KPC*–2_, *bla*_*CTX–M*–65_, *bla*_*SHV*–12_, *rmtB*, and *bla*_*TEM*–1_ was fully sequenced and characterized. To the best of our knowledge, this is the first report of conjugative pCT-KPC-like plasmid co-carrying *fosA3* and *bla*_*KPC*–2_.

## Materials and Methods

### Bacterial Strains

Fifty seven non-duplicate CRKP clinical isolates from urine (*n* = 6), sputum or bronchoalveolar lavage fluid (*n* = 35), abscess (*n* = 2), blood (*n* = 5), pus (*n* = 2), abdominal fluid (*n* = 3), and other patient samples (*n* = 6) were collected from Shandong Provincial Hospital of China, between January 2017 and June 2020. Matrix-assisted laser desorption/ionization time-of-flight mass spectrometry (MALDI-TOF/MS) (BioMérieux, Marcy-l’Étoile, France) was used to identify the isolates. Carbapenemases were detected using carbapenem inactivation method (CIM) and EDTA-modified CIM (eCIM).

### Antibiotic Susceptibility Assay

Antibiotic susceptibility was analyzed with a VITEK-2 compact system (BioMérieux, France) for aztreonam (ATM), cefepime (FEP), ceftriaxone (CRO), ceftazidime (CAZ), ertapenem (ETP), imipenem (IMP), piperacillin-tazobactam (TZP), trimethoprim-sulfamethoxazole (SXT), ciprofloxacin (CIP), levofloxacin (LVX), gentamicin (GEN), and amikacin (AMK); broth microdilution for polymyxin B (POL), tigecycline (TGC); and agar dilution for fosfomycin (FOS), using Mueller–Hinton agar supplemented with 25 μg/ml of G6P (CLSI, 2020). Susceptibility assay results were interpreted by CLSI breakpoints (CLSI, 2020), except for TGC, which were defined by the European Committee on Antimicrobial Susceptibility Testing (EUCAST, 2020) guidelines (EUCAST, 2020). Fosfomycin susceptibility was interpreted according to CLSI breakpoints for *E. coli* urinary isolates. Phenotypic detection of carbapenemases was performed using the CIM and eCIM tests (CLSI, 2020).

### Genotyping by Pulsed-Field Gel Electrophoresis

Genomic DNA from clinical strains embedded in gel plugs was digested with QuickCut *Xba*I (Takara, Shiga, Japan), and restriction fragments, ranging from 50 to 500 kb, were separated using CHEF Mapper apparatus (Bio-Rad, Hercules, CA, United States) for 19 h with the pulse time switched from 6 to 36 s. Pulsed-field gel electrophoresis (PFGE) patterns were compared using Gel-J software, version 2.0 (Heras et al., 2015). Pulsotypes were assigned to the clusters with 80% similarity (Chen et al., 2019).

### Conjugation Assay

Conjugation experiments were carried out with sodium azide-resistant *E. coli* J53Azi*^R^* being used as the recipient. Transconjugants harboring fosfomycin resistance genes were selected on Mueller–Hinton agar plates containing 64 mg/ml of fosfomycin, 100 mg/ml of sodium azide, and 25 μg/ml of G6P (Xiang et al., 2015). Transconjugants harboring carbapenemase resistance genes were selected on Mueller–Hinton agar plates containing 6 μg/ml of CAZ and 100 mg/ml of sodium azide. Antibiotic susceptibility test and PCR analysis were performed to confirm the *fosA3* and/or carbapenemase gene transfer (Poirel et al., 2011; Xiang et al., 2015). Furthermore, PCR-based replicon typing (PBRT) was used to characterize the plasmid harbored by the transconjugants (Carattoli et al., 2005).

### Whole-Genome Sequencing and Analysis

DNA from clinical isolates was extracted and sequenced using an Illumina Hiseq platform at Novogene Co., Ltd. (Beijing, China). Illumina sequences were assembled *de novo* using the SPAdes v3.10 (Nurk et al., 2013).

For the JNKPN52 and JNKPN30 isolates, genome sequencing was also performed on a PacBio RSII sequencer at Biozeron Biological Technology Co., Ltd. (Shanghai, China). The paired-end short Illumina reads were used to correct the long PacBio reads utilizing *proovread*, and then the corrected PacBio reads were assembled *de novo* utilizing SMARTdenovo.^1^ Sequence annotation was conducted using RAST 2.0^2^ combined with BLASTP/BLASTN searches against the UniProtKB/Swiss-Prot and RefSeq databases. Annotation of resistance genes and mobile elements was carried out using the online databases, including CARD^3^ and ISfinder.^4^

Antimicrobial resistance genes and multilocus sequence typing (MLST) were analyzed *in silico* by using Abricate software^5^ (Sherry et al., 2019). Virulence scores, capsular (K) serotypes, and lipopolysaccharide (LPS) O antigen serotype were predicted using Kleborate v0.3.0^6^ (Wyres et al., 2020). Single-nucleotide polymorphism (SNP) calling was performed using Snippy 3.1,^7^ and recombinant variants were excluded using ClonalFrameML 1.0 (Lu et al., 2019). Maximum likelihood phylogenetic trees were constructed with RAxML,^8^ from the recombination-free SNPs.

For our dataset, core-genome MLST (cgMLST) analysis was performed using SeqSphere+ software (8.0.2 version; Ridom, Münster, Germany) according to the ‘‘*K. pneumoniae* sensu lato cgMLST’’ version 1.0 scheme^9^ (Weber et al., 2019). A total of 2,358 target genes were used to characterize the gene-by-gene allelic profile of the *K. pneumoniae* strains. The resulting set of target genes was then used for interpreting the clonal relationship displayed in a minimum spanning tree using the “pairwise ignoring missing values” parameter during distance calculations.

### Identification of Fosfomycin-Resistant Determinants

The fosfomycin resistance-related proteins MurA, GlpT, and UhpT of the genomes were aligned with *K. pneumoniae* reference strain ATCC 700721 using local BLAST software. Multiple sequence alignments were performed by MAFFT, with the 4-kb *fosA^kp^* gene-related fragment of JNKPN10 as a reference.

### Analysis of the Plasmid Coharboring *fosA3* and *bla*_*KPC*–2_

The complete sequence of pJNKPN52_KPC_fosA has been deposited in GenBank under accession number MZ709016. Eleven fully sequenced pCT-KPC-like plasmids harboring *bla*_*KPC*–2_ were compared with pJNKPN52_KPC_fosA by BLAST Ring Image Generator,^10^ including pCT-KPC (GenBank accession no. KT185451), p69-2 (GenBank accession no. CP025458), p1068-KPC (GenBank accession no. MF168402), p20049-KPC (GenBank accession no. MF168404), p675920-1 (GenBank accession no. MF133495), pC2414-2-KPC (GenBank accession no. CP039820), pKP1034 (GenBank accession no. NZ_KP893385), pKSH203-KPC (GenBank accession no. CP034324), p16HN-263_KPC (GenBank accession no. CP045264), pEBSI036-2-KPC (GenBank accession no. MT648513), and pKP19-2029-KPC2 (GenBank accession no. CP047161). More detailed genome alignment between closely related plasmids was conducted by local BLAST and visualized with Easyfig.^11^

### Analysis of the Plasmid Harboring *bla*_*NDM*–1_

The complete sequence of pJNKPN30_NDM has been deposited in GenBank under accession number OL389795. Four fully sequenced IncA/C type plasmids harboring *bla*_*NDM*–1_ were compared with pJNKPN30_NDM by BLAST Ring Image Generator (see text footnote 10), including pNDM_KN (GenBank accession no. JN157804), pMS6198A (GenBank accession no. CP015835), p1605752AC2 (GenBank accession no. CP022126), and pT1 (GenBank accession no. KX147633).

## Results

### Antimicrobial Susceptibility

Among the 57 tested CRKP strains, 40 were resistant to fosfomycin. High resistance (> 70%) was observed against β-lactam antibiotics, fosfomycin, and quinolones. The highest resistance (> 98%) was observed against TZP, CRO, FEP, and ETP. All the strains remained 100% susceptible to TGC, and only one isolate showed resistance to POL [minimal inhibitory concentration (MIC) ≥ 64]. The antibiotic susceptibility results are shown in Table 1 and Supplementary Table 4.

**TABLE 1:** Antimicrobial resistance profile of the CRKP strains.

Antimicrobial agents	Resistance rate (%) *n* (%)	MIC_50_ (μg/ml)	MIC_90_ (μg/ml)	MIC range (μg/ml)
TZP	100	≥128	≥128	128–128
CAZ	98.2	≥64	≥64	1–64
CRO	98.2	≥64	≥64	1–64
FEP	98.2	≥64	≥64	2–64
ATM	91.2	≥64	≥64	1–64
ETP	100	≥32	≥32	2–32
IMP	87.7	≥16	≥16	1–16
AMK	57.9	≥64	≥64	2–64
GEN	73.7	≥16	≥16	1–16
CIP	87.7	≥4	≥4	0.25–4
LVX	80.7	≥8	≥8	0.25–8
SXT	54.4	≥16	≥16	1–320
POL	1.8	0.5	1	0.125–128
TGC	0	1	2	0.25–2
FOS	70.2	512	≥1,024	32–1,024

*MIC, minimal inhibitory concentrations; TZP, piperacillin/tazobactam; CAZ, ceftazidime; CRO, ceftriaxone; FEP, cefepime; ATM, aztreonam; ETP, ertapenem; IMP, imipenem; AK, amikacin; CEN, gentamicin; CIP, ciprofloxacin; LEV, levofloxacin; STX, trimethoprim/sulfamethoxazole; POL, polymyxin B; TGC, tigecycline; FOS, fosfomycin; CRKP, carbapenem-resistant Klebsiella pneumoniae.*

### Molecular Typing and Phylogenetic Group Genotyping

**FIGURE 1:**
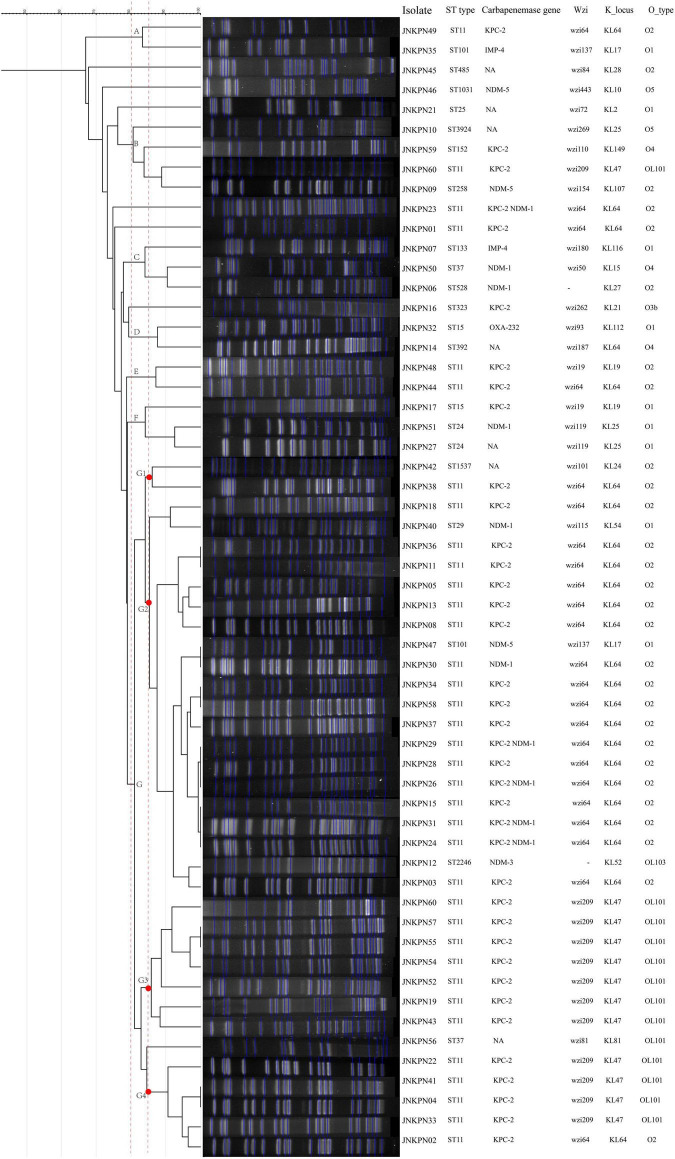
The dendrogram is based on the similarity of pulsed-field gel electrophoresis (PFGE) patterns in the 57 clinical carbapenem-resistant *Klebsiella pneumoniae* (CRKP) isolates. The right panel shows results from isolate name, sequence type, carbapenemase gene, K_locus, and O_type. NA, not available.

The 57 *K. pneumoniae* strains had 18 sequence types (STs), with ST11 being the most common (*n* = 36, 63%) (Figure 1 and Supplementary Table 2). The others were ST101 (*n* = 2), ST15 (*n* = 2), ST24 (*n* = 2), ST37 (*n* = 2), ST1031, ST133, ST152, ST1537, ST2246, ST25, ST258, ST29, ST323, ST392, ST3924, ST485, and ST528.

Among these strains, we detected 19 different K-loci, the most common being KL64 (*n* = 26, including 25 ST11) and KL47 (*n* = 10), together accounting for 63% of all the strains (Figure 1). Seven distinct O antigen encoding loci were detected among the strains, and the most common were O2 (*n* = 30), OL101 (*n* = 11), and O1 (*n* = 9).

**FIGURE 2:**
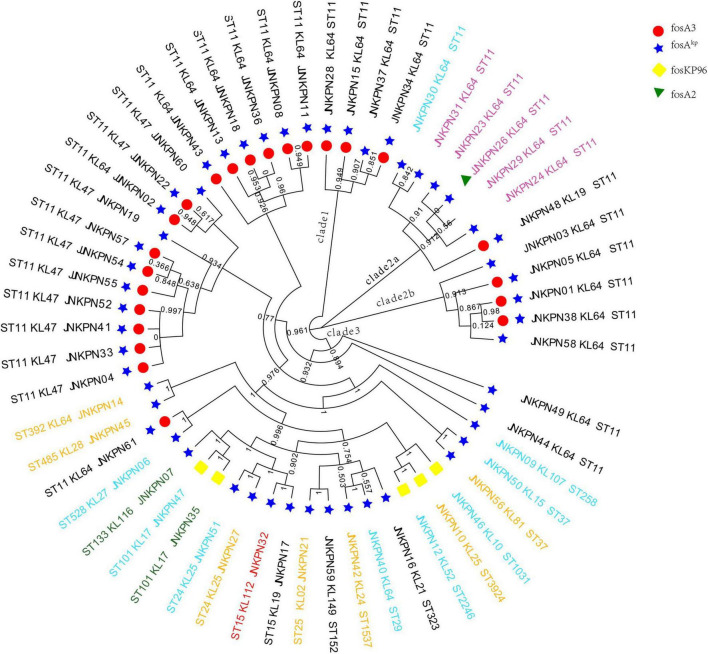
Phylogenetic analysis of carbapenem-resistant *Klebsiella pneumoniae* (CRKP) isolates. The names of isolates were colored according to the carbapenemase(s) harbored, including *bla*_*KPC*–2_ (black), *bla*_*NDM*_ (blue), *bla*_*NDM*_ and *bla*_*KPC*_ (pink), *bla*_*IMP*_ (green), and *bla*_*OXA*–232_ (red).

**FIGURE 3:**
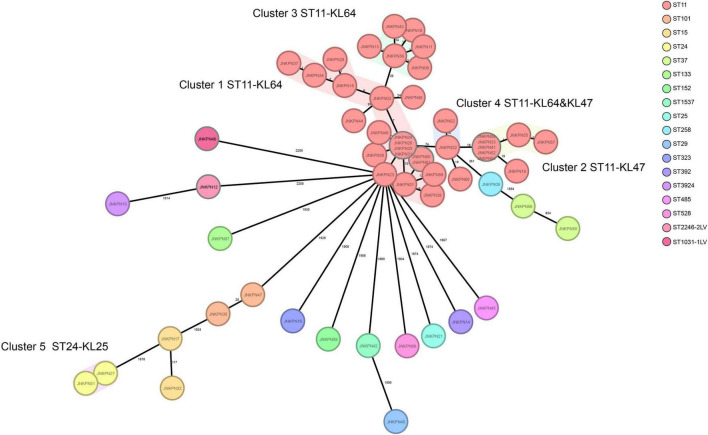
Minimum spanning tree showing core genome multilocus sequence typing (cgMLST) of the 57 carbapenem-resistant *Klebsiella pneumoniae* (CRKP) isolates, showing five cluster types numbered consecutively. Each circle represents an allelic profile. Colors of circles indicate the different sequence types (STs). Cluster types consist of closely related genotypes (≤15 allele differences). The numbers on the connecting lines illustrate the numbers of target genes with different alleles.

According to PFGE profile, seven different clusters as A∼G clone groups and five singletons were identified. The phylogenetic tree revealed that CRKP strains could be broadly clustered into three major clades: clades 1 and clades 2 consisted of ST11 strains alone, while clade 3 consisted of ST11 and the other STs (Figure 2). A minimum spanning tree of the 57 *K. pneumoniae* isolates was constructed based on cgMLST allelic profiles, showing the presence of five cluster types (≤ 15 allele differences). ST11-KL64-wzi64-O2 isolates mainly belonged to Cluster 1 (*n* = 17) and Cluster 3 (*n* = 6), while ST11-KL47-OL101 isolates mainly belonged to Cluster 2 (*n* = 7) (Figure 3 and Supplementary Table 5).

### Mechanisms of Fosfomycin Resistance

All the 57 strains harbored at least one fosfomycin-modifying enzymes, including *fosA2*, *fosA3*, *foskp96*, and *fosA^kp^* (Figure 2). *fosA^kp^* was the most prevalent chromosomal-encoded enzyme, detected in 52 isolates, followed by *foskp96*, in five isolates. *fosA^kp^* and *fosA3* were detected in 23 isolates. *foskp96* was detected among 5 isolates, though only one isolate showed fosfomycin-resistant phenotype *in vitro*.

Among the isolates, 3 isolates had Ser148Asn and Ser209Thr substitutions in MurA, and two isolates had MurA deletion. One variation, Val434Ile, in uhpT was detected in two isolates. Three substitutions in glpT, Ile260Val, Val337Ile, and Ile429Val, were detected in one isolate.

### Distribution of Antimicrobial Resistance Genes and Virulence Genes

**FIGURE 4:**
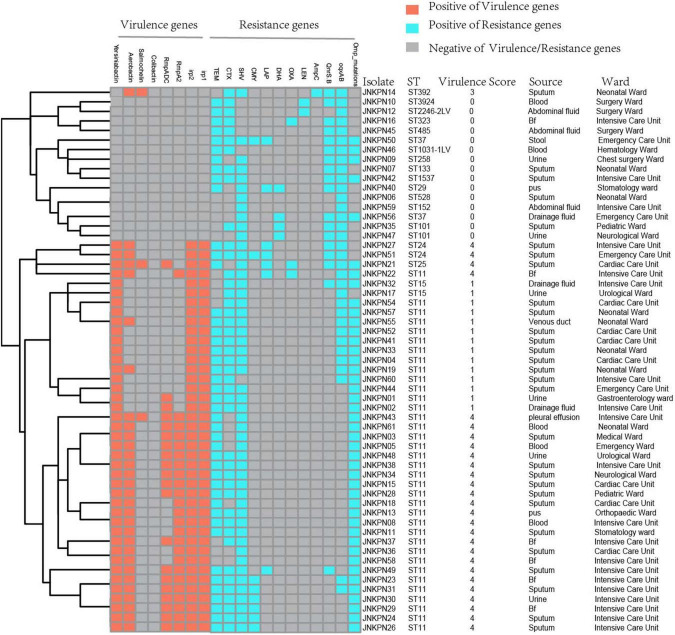
Detection of resistance genes and virulence genes in carbapenem-resistant *Klebsiella pneumoniae* (CRKP) isolates. The sequence type, virulence score, source, and ward of each isolate were marked on the right of the squares. Bf, bronchoalveolar lavage fluids.

As illustrated in Figure 1 and Supplementary Table 1, 87.7% of the isolates produced carbapenemase in accordance with the results of CIM test. *bla*_*KPC*–2_ was the main type of carbapenemase (*n* = 33). Five strains coharboring *bla*_*KPC*–2_ and *bla*_*NDM*–1_ were detected. Meanwhile, *bla*_*NDM*–1_ (*n* = 5), *bla*_*NDM*–5_ (*n* = 3), *bla*_*NDM*–3_ (*n* = 1), *bla*_*IMP*–4_ (*n* = 2), and *bla*_*OXA*–232_ (*n* = 1) also contributed to the carbapenem resistance. Twenty transconjugants harboring carbapenemase genes were acquired. One transconjugant harbored *bla*_*IMP*–4_, ten transconjugants harbored *bla*_*KPC*–2_, and nine transconjugants harbored *bla*_*NDM*_. ESBL resistance genes, such as *bla*_*TEM*_, *bla*_*CTX–M*_, *bla*_*SHV*_, *bla*_*CMY*_, and *bla*_*SHV*_, were also detected, with *bla*_*TEM*_, *bla*_*CTX–M*_, and *bla*_*SHV*_ being the most prevalent. The strains coharboring *bla*_*TEM*_, *bla*_*CTX–M*_, and *bla*_*SHV*_ made 52.6% of all the CRKP strains (Figure 4 and Supplementary Table 1).

Plasmid-encoded fluoroquinolone resistance genes (QnrS and QnrB) were detected in 18 isolates, while chromosomal *oqxA* and *oqxB* were detected in 33 isolates (Figure 4 and Supplementary Table 1). A high prevalence of specific porin defects was detected, and only 14 isolates were without any mutation in *ompK5* or *ompK36*. It was observed that *ompK35* truncations and *ompK36GD* mutations coexisted in 59.6% of the strains (Figure 4 and Supplementary Table 2).

According to the Katholt criterion, 27 isolates were assigned a virulence score of 4, which were closely related to ST11 (*n* = 24) (Figure 4). Analysis of virulence genes showed that 41 isolates possessed yersiniabactin genes located on ICEKp3 (*n* = 38), ICEKp1 (*n* = 1), ICEKp5 (*n* = 1), and ICEKp12 (*n* = 1), with ICEKp3 being the most prevalent carrier. Of 25 ST11-KL64 isolates, 22 (88%) carried *rmpA2* gene, but only three were positive for the string test, suggesting that *rmpA2* was inactive in most isolates. Among 10 ST11-KL47 isolates, only one was positive for *rmpA2* gene (Supplementary Table 2).

### Genetic Background of *fosA* in Carbapenem-Resistant *Klebsiella pneumoniae*

Four types of genetic environments existed in the 52 *fosA^kp^*-positive CRKP isolates. The upstream genes of *fosA^kp^* among the strains are identical, but those downstream are variable. The intergenic regions between *fosA^kp^* and the downstream MocR gene could be DNA helicase-related genes, a Type I restriction–modification system, or a hypothetical Protein. As shown in Supplementary Figure 1, the genetic environment of *foskp96* was similar to that of *fosA^kp^*, with a backbone of *YrkL-LysR-FosA-MocR-YjiS*. The genetic environment of *fosA3* was consistent, where *fosA3* is flanked by *IS26* at both ends, in a transposon-like structure.

### Phenotypic and Genotypic Characteristics of Plasmids Harboring *fosA3* and *bla*_*KPC*–2_

The plasmids harboring *fosA3* from strains JNKPN52, JNKPN54, JNKPN55, and JNKPN57 were successfully transferred into *E. coli* J53Azi*^R^* by conjugation. All the four transconjugants were resistant to CRO, FEP, CAZ, ATM, TZP, ETP, IMP, AMK, GEN, and FOS but were susceptible to SXT, CIP, LVX, TGC, and POL (Supplementary Table 3). JNKPN52 and JNKPN54 were isolated from 2-month-old pediatric patients after cardiac surgery enrolled in the cardiac care unit in December 2019. JNKPN55 and JNKPN57 were isolated from premature babies enrolled in the neonatal intensive care unit in December 2019. A nosocomial outbreak caused by a clone of ST-KL47 KPC-KP strains was considered according to the high similarities of PFGE patterns of group G3 (> 85%) (Figure 1) and the high level of correlation within cgMLST Cluster 2 (up to 1 allele difference) (Figure 3).

**FIGURE 5:**
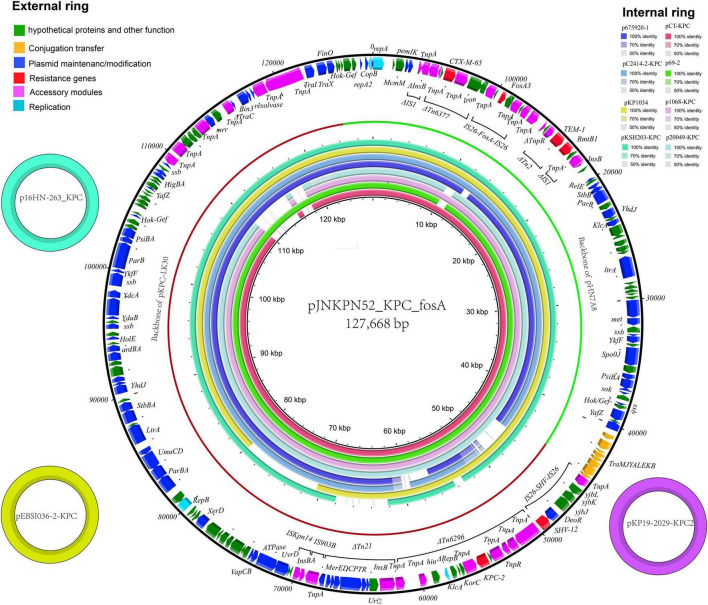
The external ring is the main structural feature of plasmid pJNKPN52_KPC_fosA (GenBank accession no. MZ709016). The three small external rings display plasmids coharboring *bla*_*KPC*–2_ and *fosA* with identity > 99%. The internal eight rings showed the comparative analysis of *bla*_*KPC*_-harboring plasmids with pJNKPN52_KPC_fosA (constructed by BRIG).

The complete sequence of plasmid pJNKPN52_KPC_fosA from clinical strain JNKPN52 was determined to better characterize the self-transmissible plasmid coharboring *fosA3* and *bla*_*KPC*–2_. pJNKPN52_KPC_fosA is a 127,668-base pair (bp) multireplicon plasmid that belongs to the IncR:IncFII-type and shares a similar structure with pHN7A8/pKPC-LK30 hybrid plasmid pCT-KPC (Figure 5).

pJNKPN52_KPC_fosA contains two major accessory resistance regions, including the *bla*_*KPC*–2_ region harboring *bla*_*KPC*–2_ and *bla*_*SHV*–12_, and the multidrug-resistant (MDR) region carrying *rmtB* (aminoglycoside resistance), *fosA3*, *bla*_*TEM*–1_, and *bla*_*CTX–M*–65_. The MDR region was generated from the insertion of ΔTn6377–*bla*_*CTX–M*–65_, *IS26–fosA3–IS26* unit, ΔTn2-*rmtB* element within *IS1*. The *bla*_*KPC*–2_ region was organized in order of a truncated *IS26–bla*_*SHV*–12_–*IS26* unit, ΔTn6296, ΔTn21 with insertion of *IS5075*, an *IS903B* remnant, and *ISKpn14* (Figure 5).

### Comparative Analysis of Plasmids Harboring *fosA3–bla*_*KPC*–2_

According to sequence alignment by BRIG, pJNKPN52_KPC_fosA showed 99% nucleotide identity with the previously reported plasmids p16HN-263_KPC and pKP19-2029-KPC2 isolated from China and pEBSI036-2-KPC isolated from Egypt (Ahmed et al., 2021) (Figure 5).

**FIGURE 6:**
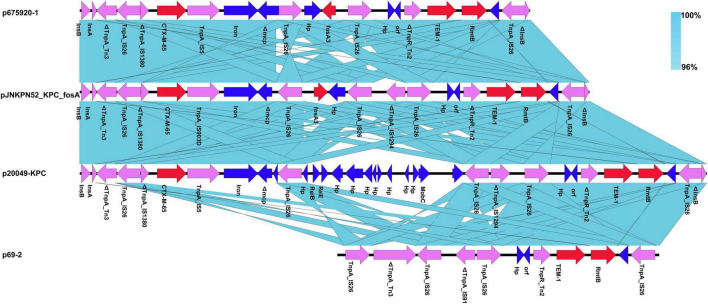
Comparison of genetic context of the multidrug-resistant (MDR) region with related regions.

The MDR regions of the eleven plasmids were similar, with pJNKPN52_KPC_fosA, p675920-1, p69-2, and p20049-KPC slightly differing from one another. To determine the detailed structural differences between these plasmids, additional linear comparative genomics analysis was performed by BLAST. Compared with pJNKPN52_KPC_fosA, p675920-1 lacked a Δ*IS1294* region, possibly because of recombination of *IS26–fosA3–IS26* region. In p20049-KPC, the deletion of *IS26–fosA3–IS26* region and insertion of partial plasmid backbone genes were observed within the MDR region (Figure 6).

**FIGURE 7:**
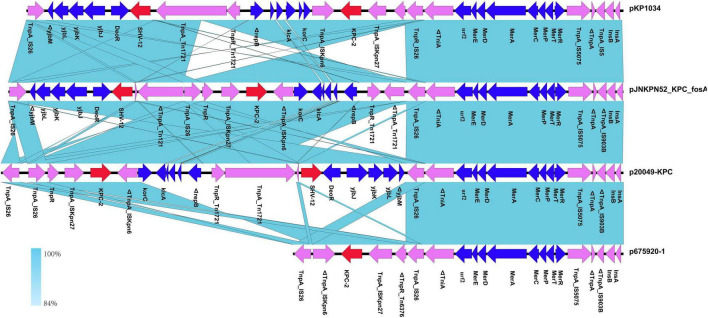
Comparison of genetic context of the *bla*_*KPC*–2_ region with related regions.

The *bla*_*KPC*–2_ region of the pJNKPN52_KPC_fosA was similar to that of p20049-KPC and pKP1034, with the inversion of ΔTn6296. Compared with pJNKPN52_KPC_fosA, the deletion of the truncated *IS26–bla*_*SHV*–12_–*IS26* unit was observed in p675920-1, probably due to *IS26*-mediated deletion (Figure 7).

### Genotypic Characteristics of Plasmids Harboring *bla*_*NDM*–1_

The plasmids harboring *bla*_*NDM*_ from strains JNKPN23, JNKPN24, JNKPN26, JNKPN29, JNKPN30, JNKPN31, JNKPN46, JNKPN47, and JNKPN51 were successfully transferred into *E. coli* J53Azi*^R^* by conjugation. All the nine transconjugants were resistant to CRO, CAZ, TZP, ETP, and IMP, but were susceptible to FOS, CIP, and LVX (Supplementary Table 6). All the plasmids harboring *bla*_*NDM*–1_ were shown to belong to IncA/C2 type through plasmid typing (Supplementary Table 6).

**FIGURE 8:**
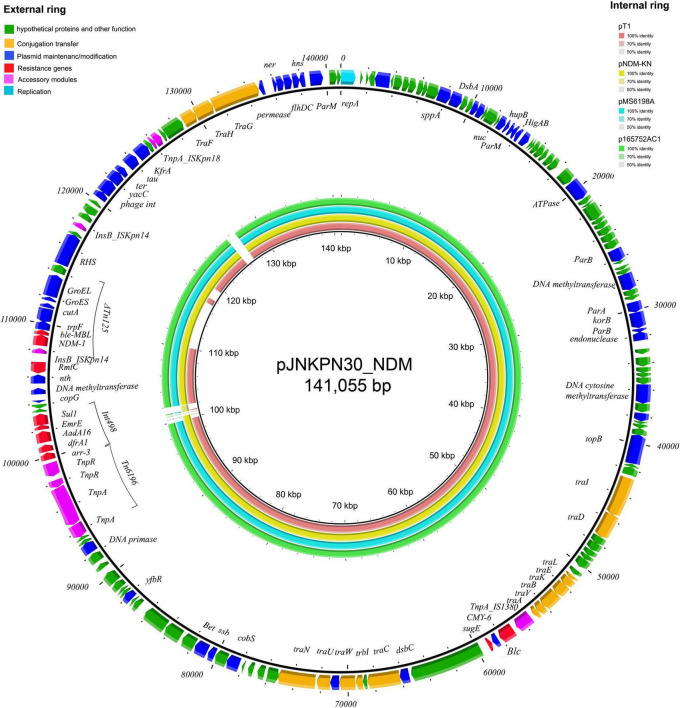
The external ring is the main structural feature of plasmid pJNKPN30_NDM (GenBank accession no. OL389795). The internal four rings showed the comparative analysis of *bla*_*NDM*_-harboring plasmids with pJNKPN30_NDM (constructed by BRIG).

The complete sequence of plasmid pJNKPN30_NDM from clinical strain JNKPN30 was determined to better characterize the self-transmissible IncA/C2 type plasmid harboring *bla*_*NDM*–1_. pJNKPN30_NDM contains two main accessory resistance regions, including the *IS1380*–*bla*_*CMY*–6_ region and the MDR region carrying *Tn6196*, class 1 integron structure bearing *arr-3*, *dfrA1*, *AadA16*, *ErmE*, and *sul1*, and partial of Tn125-bearing *bla*_*NDM*–1_ interrupted by the insertion of Δ*ISKpn14*, followed by the 16S rRNA methylase rmtC gene (Figure 8).

According to sequence alignment by BRIG, pJNKPN30_NDM was with a backbone similar to that of other IncA/C2 plasmids (84–99% query coverage, > 99% nucleotide sequence identity). The main regions of discontinuity were within the *Int498* region and the *ISKpn18* region (Figure 8).

### Nucleotide Sequence Accession Numbers

Raw reads of all 57 isolates have been deposited in GenBank (BioProject PRJNA769451). The complete sequence of pJNKPN52_KPC_fosA and pJNKPN30_NDM has been deposited in GenBank under accession numbers MZ709016 and OL389795, respectively.

## Discussion

The emergence of CRKP has become a crucial public health problem, as it limits treatment options and requires novel active agents or combination therapies (Ekwanzala et al., 2019). *K. pneumoniae* isolates have shown susceptibility to fosfomycin; hence, the “old” antibiotic agent is being re-considered as a possible auxiliary drug (Vardakas et al., 2016). Pontikis et al. (2014) reported that intravenous fosfomycin for nosocomial CRKP infections has a good clinical outcome. Intravenous fosfomycin is used in many countries and has completed phase 3 clinical trials for the treatment of urinary tract infection and acute pyelonephritis (Ito et al., 2017). However, information on resistance to fosfomycin among CRKP in China is inadequate. The occurrence of fosfomycin-resistant CRKP in China ranges from 18.7 to 80% (Tseng et al., 2017; Huang et al., 2021). These inconsistent data indicate that resistance of *K. pneumoniae* isolates to fosfomycin, especially to CRKP, requires further evaluation. Our results indicated that 70.2% of CRKP strains were resistant to fosfomycin in Shandong, which is much higher than that in most regions in China.

Fosfomycin resistance may be due to chromosome-encoded *murA*, *glpT*, *uhpT*, *uhpA*, *ptsI*, and *cyaA* mutations, or plasmid-encoded or chromosomal inactivation by fosfomycin-modifying enzymes (Liu et al., 2020). We found that *fosA* homologs were widely distributed among the CRKP strains, and all the strains harbored *fosA^kp^* or *foskp96*. Moreover, 40.3% of the strains had both *fosA^kp^* and *fosA3*. It was difficult to discriminate *fosA* variants located on plasmids from those on chromosomes because the analysis was based on draft sequences of genomes. *fosA^kp^* or *foskp96* is intrinsically distributed on *K. pneumoniae* chromosomes (Ito et al., 2017). According to the previous report, MIC_50/90_ values of fosfomycin for *K. pneumoniae* clinical strains producing KPC-type carbapenemase were 16/64 μg/ml (Ito et al., 2017). In this study, 34 CRKP isolates without *fosA3* had a MIC range of ≤ 32 to ≥ 1,024 μg/ml and MIC_50/90_ values of 256/1,024 μg/ml. Not all the isolates showed fosfomycin resistance. We speculated that the dissimilarity between fosfomycin-resistant genotype and phenotype was caused by the expression level of *fosA* gene.

*fosA3* is the most common acquired *fosA*, encoded by plasmids (Ito et al., 2017). Apart from *fosA* production, fosfomycin resistance is also related to *MurA*, *glpT*, and *uhpT* mutations, which were rare in our isolates, and only 2, 3, and 1 isolates, respectively, were detected. Therefore, our results strongly suggest that *fosA3* and *fosA* contribute mainly to fosfomycin resistance.

Further, pLVPK-like-positive ST11-KL64 isolates show better survival in the environment (Zhou et al., 2020). Fourteen *fosA3*-positive strains were screened from the ST11-KL64-wzi64-O2 subgroup, and eight *fosA3*-positive strains were screened from the ST11-KL47-wzi209-OL101 subgroup. Twenty-two out of 25 ST11-KL64 strains and only one ST11-KL47 strain contained *rmpA2*. All the seven isolates belonging to cgMLST Cluster 2 and Cluster 6 isolates belonging to cgMLST Cluster 3 coharbored *bla*_*KPC*–2_ and *fosA3*. The isolates belonging to cgMLST Cluster 1 and Cluster 3 except JNKPN01 and JNKPN30 were positive for *bla*_*KPC*–2_ and *rmpA2*. PFGE profiles and cgMLST confirmed that the clonal relation may predominantly be due to clonal dissemination.

It was noteworthy that *bla*_*NDM*_-producing CRKP isolates were increasingly reported (Qamar et al., 2021). In this study, 24.6% (14/57) of CRKP isolates were positive for *bla*_*NDM*_ (including the isolates co-producing *bla*_*KPC*–2_ and *bla*_*NDM*_), which were higher than that in the previous research in China (11.5%) (Wang et al., 2018). Plasmids harboring *bla*_*NDM*_ from 64.3% (9/14) of *bla*_*NDM*_-carrying isolates could be transferred to the recipients. The conjugative IncA/C2 type plasmids played an important role in the rapid and efficient dissemination of the *bla*_*NDM*–1_ gene among CRKP isolates in this study. According to the previous report, IncA/C type plasmids were known to be of broad host range and had been detected in numerous MDR Gram-negative species (Carattoli et al., 2012). Moreover, an outbreak caused by a clone of *Citrobacter freundii* strains bearing *bla*_*NDM*–1_ located on IncA/C plasmids and secondary *in vivo* spread of an IncA/C2 plasmid with *bla*_*NDM*–1_ to *E. coli*, *K. pneumoniae*, and *Klebsiella oxytoca* from the individual patients was reported in Denmark (Hammerum et al., 2016). Thus, more attention should be paid to monitoring and controlling the horizontal transmission of *bla*_*NDM*_ mediated by IncA/C type plasmids among *K. pneumoniae* isolates.

Interestingly, *fosA3* was not detected in any CRKP strains due to class B carbapenemase, including *bla*_*IMP*_ or *bla*_*NDM*_. According to the MLST results, KPC-2-producing ST11 was the only clone in our study closely related to *fosA3*. Analysis of the genetic environment confirmed that the mobile element *IS26–fosA3–IS26* played an important role in the dissemination of fosfomycin resistance. Moreover, most of the *fosA3*-positive KPC-2 producing strains (22/23) carried at least two kinds of ESBLs, indicating that ST11 type *K. pneumoniae* might be a good reservoir of resistance genes.

According to previous research, the high prevalence of fosfomycin resistance in KPC-producing isolates from China is associated with plasmids coharboring *fosA3* and *bla*_*KPC*_ (Singkham-In et al., 2020). Recently, *fosA3* and *bla*_*KPC*–2_ genes located on non-conjugative pCT-KPC-like plasmids have been sporadically reported in China (Xiang et al., 2015; Liu et al., 2018; Shi et al., 2018; Zhai et al., 2021). Furthermore, Shi et al. (2018) proved that *bla*_*KPC*–2_- and *rmtB*-carrying pCT-KPC-like plasmids were prevalent among clonal *K. pneumoniae* CG258 strains collected from five different hospitals and were associated with the dissemination of *bla*_*KPC*–2_ and *rmtB*. It seemed that the spread of the pCT-KPC-like plasmids was mainly due to the clonal dissemination of ST11 KPC-producing *K. pneumoniae*, as the conjugation tests failed to recover transconjugants in all reports (Zhai et al., 2021).

Recently, a plasmid pEBSI036-2-KPC from a high-risk clone ST11 KL47 serotype of a CR-HvKP strain isolated from an Egyptian hospital was reported. pJNKPN52_KPC_fosA showed 99% identity with pEBSI036-2-KPC. But the transferability of plasmid pEBSI036-2-KPC was not determined (Ahmed et al., 2021). In our study, four ST11-KL47 type *K. pneumoniae* strains, JNKPN52, JNKPN54, JNKPN55, and JNKPN57, could co-transfer *fosA3* and *bla*_*KPC*–2_ genes into recipient *E. coli* J53Azi*^R^* by conjugation tests, indicating that the *fosA3* gene could be co-disseminated with *bla*_*KPC*–2_. The four strains isolated from different patients were identified from the same clone according to the PFGE profiles and cgMLST cluster. Notably, *bla*_*KPC*–2_ and *fosA3* genes were confirmed to be located on a plasmid pJNKPN52_KPC_fosA, which shared similar backbones with the previously reported pCT-KPC-like plasmids, including pKP1034, pCT-KPC, pKPC-LK30, p69-2, and p675920-1. For the first time, we confirmed the pCT-KPC-like plasmid-mediated horizontal transmission of *bla*_*KPC*–2_ and *fosA3* resistance. We noticed that ten copies of *IS26* were detected in plasmid pJNKPN52_KPC_fosA and would mediate homologous recombination and mobilization of accessory resistance regions within and among different plasmids (Zhai et al., 2021). Therefore, *IS26* may have played a vital role in the generation process of pJNKPN52_KPC_fosA.

## Conclusion

Our findings indicate that *fosA* is intrinsically distributed in the genome of clinically isolated *K. pneumoniae* and might contribute to fosfomycin resistance. The coexistence of plasmid-mediated *fosA3* and chromosomal-encoded *fosA^kp^* was observed commonly among ST11 CRKP strains. The emerging conjugative pCT-KPC-like plasmids coharboring *bla*_*KPC*–2_ and *fosA3* would exacerbate the fosfomycin resistance among CRKP strains. ST11-KL64 and ST11-KL47 *K. pneumoniae*, the so-called “super-bug,” with higher resistance and virulence, should be monitored by more effective strategies to prevent the future dissemination of resistance.

## Data Availability Statement

The datasets presented in this study can be found in online repositories. The names of the repository/repositories and accession number(s) can be found in the article/Supplementary Material.

## Author Contributions

YW contributed to experiment conception and design. YH, YZ, and RC conducted bioinformatics analysis and wrote the manuscript. XZ and YB performed the data analysis. ZS carried out the bacteria identification. XL and CZ prepared the tables and figures. YW is responsible for submitting a competing interest statement on behalf of all authors of the manuscript. All authors contributed to the article and approved the submitted version.

## Conflict of Interest

The authors declare that the research was conducted in the absence of any commercial or financial relationships that could be construed as a potential conflict of interest.

## Publisher’s Note

All claims expressed in this article are solely those of the authors and do not necessarily represent those of their affiliated organizations, or those of the publisher, the editors and the reviewers. Any product that may be evaluated in this article, or claim that may be made by its manufacturer, is not guaranteed or endorsed by the publisher.

## Footnotes

1
https://github.com/ruanjue/smartdenovo

2
https://rast.nmpdr.org/

3
http://arpcard.mcmaster.ca

4
https://www-is.biotoul.fr/

5
https://github.com/tseemann/abricate

6
https://github.com/katholt/Kleborate

7
https://github.com/tseemann/snippy

8
https://github.com/stamatak/standard-RAxML

9
https://www.cgmlst.org/ncs/schema/2187931/

10
https://sourceforge.net/projects/brig/

11
http://mjsull.github.io/Easyfig/files.html

## Supplementary Material

Supplementary Figure 1 Comparison of genetic environments of the *fosA*. **(A)** The genetic environment of *fosA**^kp^* and *foskp96*; **(B)** The genetic environment of *fosA2*; **(C)** The genetic environment of *fosA3*.
