# Supplementary material for: Clonal Dissemination of Clinical Carbapenem-Resistant Klebsiella pneumoniae Isolates Carrying fosA3 and blaKPC–2 Coharboring Plasmids in Shandong, China
Source: Front Microbiol. 2021 Dec 17;12:771170. doi: 10.3389/fmicb.2021.771170 (PMC8718808; doi:10.3389/fmicb.2021.771170)
Supplement: Supplementary file 4 [file Table_3.doc]

| MIC (μg/mL) | | | | | | | | | | | | | | | |
| --- | --- | --- | --- | --- | --- | --- | --- | --- | --- | --- | --- | --- | --- | --- | --- |
| Isolates | CRO | FEP | CAZ | ATM | TZP | ETP | IMP | SXT | AMK | GEN | CIP | LEV | FOS | TGC | POL |
| JNKPN52 | >64 | >64 | >64 | >64 | >128 | >32 | >16 | 1/19 | >64 | >16 | >4 | >8 | >1024 | 0.5 | 1 |
| J52 | **>64** | **64** | **16** | **>64** | **>128** | **32** | **16** | **1/19** | **>64** | **>16** | **0.12** | **0.12** | **>1024** | **0.5** | **0.5** |
| JNKPN54 | >64 | >64 | >64 | >64 | >128 | >32 | >16 | 1/19 | >64 | >16 | >4 | >8 | >1024 | 0.5 | 1 |
| J54 | **>64** | **64** | **64** | **>64** | **>128** | **32** | **8** | **1/19** | **>64** | **>16** | **0.12** | **0.12** | **>1024** | **0.5** | **0.5** |
| JNKPN55 | >64 | >64 | >64 | >64 | >128 | >32 | >16 | 1/19 | >64 | >16 | >4 | >8 | >1024 | 0.5 | 1 |
| J55 | **>64** | **64** | **32** | **>64** | **>128** | **32** | **8** | **1/19** | **>64** | **>16** | **0.12** | **0.12** | **>1024** | **0.5** | **0.5** |
| JNKPN57 | >64 | >64 | >64 | >64 | >128 | >32 | >16 | 1/19 | >64 | >16 | >4 | >8 | >1024 | 0.5 | 1 |
| J57 | **>64** | **64** | **>64** | **>64** | **>128** | **32** | **8** | **1/19** | **>64** | **>16** | **0.12** | **0.12** | **>1024** | **0.5** | **0.5** |
| J53Azi^R^ | <=0.25 | <=0.12 | <=0.12 | <=1 | <=4 | <=0.12 | <=0.25 | <=**1/19** | <=2 | <=0.5 | <=0.25 | <=0.12 | <=32 | **0.5** | **0.5** |

**TABLE S3** | Antimicrobial resistance profiles of CRKP strains carrying *fosA3* and *bla*_KPC-2_ and their transconjugants.

MIC, minimal inhibitory concentrations; CRO, ceftriaxone; FEP, cefepime; CAZ, ceftazidime; ATM, aztreonam; TZP, piperacillin-tazobactam; ETP, ertapenem; IMP, imipenem; SXT, trimethoprim-sulfamethoxazole; AMK, amikacin; GEN, gentamicin; CIP, ciprofloxacin; LVX, Levofloxacin; FOS, fosfomycin; TGC, tigecycline; POL, polymyxin B.
